# OLDER ITALIANS’ VIEWS OF AND EXPERIENCES WITH IMMIGRANT CAREWORKERS

**DOI:** 10.1093/geroni/igz038.2585

**Published:** 2019-11-08

**Authors:** Katia Vecchione, Anne Barrett

**Affiliations:** 1 University of Trento, Trento, Italy; 2 Florida State University, Tallahassee, Florida, United States

## Abstract

Population aging has led, in many countries, to new care arrangements to meet the growing need. In Italy, with the second oldest population in the world, family members, especially women, provide the majority of care; however, paid immigrants are increasingly filling in where families cannot. Known as “badanti,” most of these careworkers are middle-aged women from Eastern Europe. Although some research examines this phenomenon, it focuses exclusively on careworkers – not those receiving their care. Addressing this gap, my paper examines older Italians’ attitudes toward and experiences with immigrant careworkers, using interviews with 20 nursing home residents and 20 senior center participants. Analyses reveal polarized views of “badanti, with more positive views found among those with personal experience receiving their care. I find that negative attitudes are shaped by three broader cultural discourses about aging, as well as immigration. Attitudes are influenced by views of independence and autonomy -- core values perceived to be threatened by badanti. Relatedly, attitudes are influenced by the centrality of space and home, which again are viewed as challenged by badanti’s presence. Negative views of badanti also are shaped by dominant discourses regarding immigrants, who are viewed as threats to security, particularly regarding one’s belongings. Such beliefs and values influence older adults’ willingness to accept help from careworkers and its effectiveness -- knowledge of which can help create better care scenarios.

